# Transmission and full-band coherent detection of polarization-multiplexed all-optical Nyquist signals generated by *Sinc*-shaped Nyquist pulses

**DOI:** 10.1038/srep13649

**Published:** 2015-09-01

**Authors:** Junwen Zhang, Jianjun Yu, Nan Chi

**Affiliations:** 1 Department of Communication Science and Engineering, and Key Laboratory for Information Science of Electromagnetic Waves (MoE), Fudan University, 220 Handan Road, Shanghai 200433, China; 2 ZTE (TX) Inc, Morristown, NJ 07960, USA

## Abstract

All optical method is considered as a promising technique for high symbol rate Nyquist signal generation, which has attracted a lot of research interests for high spectral-efficiency and high-capacity optical communication system. In this paper, we extend our previous work and report the fully experimental demonstration of polarization-division multiplexed (PDM) all-optical Nyquist signal generation based on *Sinc*-shaped Nyquist pulse with advanced modulation formats, fiber-transmission and single-receiver full-band coherent detection. Using this scheme, we have successfully demonstrated the generation, fiber transmission and single-receiver full-band coherent detection of all-optical Nyquist PDM-QPSK and PDM-16QAM signals up to 125-GBaud. 1-Tb/s single-carrier PDM-16QAM signal generation and full-band coherent detection is realized, which shows the advantage and feasibility of the single-carrier all-optical Nyquist signals.

Recently, high-spectral-efficiency technologies are attracting more and more research interests in optical transmission system, considering the bandwidth limitation and the requirement of cost reduction per bit per Hz^1^,^2^,^3^,^4^,^5^,^6^,^7^,^8^,^9^,^10^,^11^,^12^,^13^,^14^,^15^. Orthogonal multiplexing technologies, known as OFDM and Nyquist pulse multiplexing, are good solutions to form the data in the minimum bandwidth^1^,^2^,^3^,^4^,^5^,^6^,^7^,^8^,^9^,^10^,^11^,^12^,^13^,^14^,^15^,^16^. Until now, electrical and all-optical OFDM signal generation with high-order modulation formats has been widely investigated^1^,^2^,^3^,^4^. Compared with OFDM, Nyquist signals have several unique advantages such as lower receiver complexity, lower receiver bandwidths and lower peak-to-average power ratios^4^,^5^,^6^,^7^,^8^. In previous works, the Nyquist signal generation can be realized either by electrical Nyquist shaping^4^,^5^,^6^,^7^,^8^ or optical pulse shaping method^9^,^10^,^11^,^12^. In^5^, they have demonstrated high transmission capacity with high spectrum efficiency by the single-laser 32.5 Tbit/s Nyquist WDM system based on 325 optical carriers with 12.5GBaud PDM-16QAM signal. However, the Nyquist pulse-shaping methods for each carrier reported in^5^,^6^,^7^,^8^ are realized by the electronics digital filters with limited sampling rate and limited processor capacities. Otherwise, to generate the high baud signals, multiple spectral slices synthesis multiplexing technique is proposed based on transmitter side digital signal processing (DSP). However, it requires complex digital signal processing (DSP) for spectral slices operation and baseband down conversions^13^,^14^.

Alternatively, all optical method is considered as a promising technique for high symbol rate Nyquist signal generation^9^,^10^,^11^,^12^,^15^,^16^. There have been several reports of all optical Nyquist signals generation. In^9^, multiple optical tapped-delay-lines are proposed to generate the Nyquist signals similar as digital FIR filters. However, until now only 32-GBaud QPSK signal is generated and the requirement of multiple pumps and sources makes the scheme more complicated for high speed signals generation. On the other hand, schemes based on optical Nyquist filtering^10^ or a liquid crystal spatial modulator to form a raised-cosine Nyquist pulse shaping^11^ have also been reported. However, the filtered optical-Nyquist-pulse generation produces much higher roll-off factors, such as 0.5^10^,^11^; therefore, multiplexing using this kind of pulses results in a non-optimal use of bandwidth. In^12^, 107GBaud optical filtered Nyquist signals are generated and detected; however, only back to back and single polarization results are reported.

In most recent work of^15^, a novel all-optical method to produce sinc-shaped Nyquist pulses of very high quality has been proposed based on the direct synthesis of a rectangular shaped and phase-locked frequency comb. Using this method, in^16^ we have demonstrated the first experimental demonstration of the 125-GBaud all-optical Nyquist single polarization QPSK signal generation and full-band signal coherent detection with one receiver. Meanwhile, the previous highest ETDM symbol rate for one coherent receiver for full-band signal detection is 107Gbaud in^17^,^18^ and 110GBaud in^19^. However, only single polarization back to back performances of 75 and 125-GBaud all optical Nyquist signal is measured without transmission^16^. The performances of high-order QAM such as PDM-16QAM signals are not considered.

Therefore, in this paper, we extend our work and report the fully experimental demonstration of polarization-division multiplexed (PDM) all-optical Nyquist signal generation, fiber-transmission and digital full-band coherent detection. The PDM Nyquist signals are fully-band detected as one signal using one high speed digital coherent receiver with a continuous-wave (CW) free-running local oscillator (LO). Finally, we have successfully demonstrated the generation, transmission and full-band coherent detection of all-optical Nyquist PDM-QPSK and PDM-16QAM signals up to 125-GBaud. The back to back and transmission performances of the generated all optical Nyquist PDM-QPSK and 16QAM signals at different symbol rates are measured.

Figure 1(a) shows the basic concept of the time-frequency correspondence of the Sinc-shaped Nyquist pulses and the modulated Nyquist signals, and Fig. 1(b) shows the principle of PDM all-optical Nyquist signal generation, modulation, multiplexing and digital full-band coherent detection. As shown in Fig. 1(a), the spectrum of a single Sinc pluse is rectangular in frequency domain, while the spectrum of a Sinc-shaped pulse sequence is a frequency-locked comb with equal amplitude and the phases of the all frequency components are locked showing a linear dependence on frequency. As analyzed in^15^,^16^, a promising way of Nyquist signal generation is based on these combs and orthogonal optical time-division multiplexing (orth-OTDM)^9^. Figure 1 shows an *N*-tone frequency-locked comb with central frequency *f*_*0*_, carrier frequency-spacing of *Δf*. The pulse repetition period is represented as *T* = *1/Δf*. For an *N* tone frequency-locked comb with equal phase and amplitude, the time domain optical signal is a periodic Sinc-shaped Nyquist pulse with period of *T*. Therefore, instead of shaping the Sinc pulses, the target is to generate a frequency-locked optical comb with equal amplitude and linear-locked phase.

After Sinc-shaped Nyqust pulses generation, the Nyquist pulses can be modulated and multiplexed in the time-domain using the orth-OTDM method. PDM is also compatible with the orth-OTDM as shown in Fig. 1(b). Since the zero-crossing pulse duration is *2/(NΔf)* = *2T/N*, the periodic Sinc-shaped Nyquist pulse sequence is then split into *N* branches for independent modulation in each polarization with accurate delay of *t* = *nT/N* and *n* = *0* ~ *N* − *1*. In this way, each pulse can be multiplexed to the zero-crossing time slots of the adjacent pulses with zero ISI for each polarization state. After PDM, a modulated Nyquist signal with symbol rate of *B* = *NΔf* can be obtained. As analyzed in^15^,^16^, the spectrum of the modulated Sinc-shaped pulses is given by the convolution of the frequency comb as shown in Fig. 1. The combined signals can be treated as one entire Nyquist signal for fiber transmission.

After fiber transmission, at the coherent receiver, one free-running CW-LO working at the center frequency of the comb is used for full-band coherent detection. The full-band coherent receiver is simpler compared with the typical OTDM receiver^20^,^21^. Instead of using a large number of receivers of smaller bandwidth, we use one receiver with wider bandwidth and simplified structures^16^. On the other hand, as shown in^20^,^21^, in order to get the right time slot, the receiver-side LO should be strictly synchronized-pulse source. However, full-band coherent detection with digital signal processing (DSP) enables the digital domain time demultiplexing with simple algorithms. Therefore, the LO can be commercially available, free-running, CW-laser, which is quite easy for implementation. With regular algorithms commonly used in coherent system^22^,^23^ and digital time demultiplexing, one can recovery the all-optical Nyquist signals after the DSP. It is worth noting that, for the multiplexed signals at higher symbol rate, larger bandwidth ADCs and PDs are required.

## Results

As a proof of concept, Fig. 2 shows the experiment setup of PDM all-optical Nyquist signal generation based on *Sinc*-shaped Nyquist pulse, fiber transmission and full-band coherent detection. As proposed and demonstrated in^15^, this kind of Sinc-shaped pulse source can be realized by the cascaded Mach–Zehnder modulators (MZM) driven by the radio frequency (RF) signals, of which the RF driving voltage and direct-current (DC) bias are properly chosen. Similar experiment setup with transmitter and coherent receiver can be found in our previous work^16^. One external cavity laser (ECL) is used as the light source at 1549.50 nm, linewidth less than 100 kHz and output power of 14.5 dBm. The frequency-locked optical comb with 12.5 and 25-GHz carrier spacing is generated by a MZM driven by the RF source at 12.5 and 25 GHz, respectively. One electrical amplifier (EA) is used to boost the amplitude of the RF signal. In our case, we choose the driving voltage at about 2Vpi and the DC bias at about 0.52Vpi and Vpi is half-wave voltage of the modulator^16^. One polarization-maintaining tunable optical filter (PM-TOF) is used to choose the comb tones. Using different frequency RF sources and different bandwidth PM-TOF, we can generate different combs with different carrier spacing and different Nyquist pulses with different repeating period.

### 12.5 GHz spacing combs and Nyquist pulses

We first use the RF source with frequency at 12.5 GHz to generate the combs. Figure 3(a,b) show the 3 × 12.5-GHz and 5 × 12.5-GHz optical comb after PM-TOF with different filtering bandwidth. The power difference of the generated 3-tone and 5-tone combs after the filter is less than 0.3 dB. The phases of the comb tones are locked showing a linear dependence on frequency. In the time domain, the waveforms of the 3-tone and 5-tone combs are shown in Fig. 3(c,d) as Sinc-shaped pulses. We can see, for the 3 × 12.5-GHz comb, the Sinc-shaped Nyquist pulses obtained have two zero-crossing points between each Nyquist pulse and the pulse repeating period is 80 ps. The repeating period is also 80 ps for the 5 × 12.5 GHz comb. However, the Nyquist pulses have four zero-crossing points between each pulse. The pulse durations between two zero-crossings in (c) and (d) are 53.33 and 32 ps, respectively. The optical spectra of the signals are all measured by a commercial optical spectrum analyzer (Yokogawa, AQ6370B). The time-domain waveforms of the Nyquist pulse and multiplexed signals are all measured by an optical sampling oscilloscope (Agilent 86116B) with 65 GHz bandwidth.

For Nyquist signal generation, we first modulate the Sinc-shaped Nyquist pulse and then multiplex these time-division channels by polarization-maintaining optical coupler (PM-OC) and tunable optical delay-line (PM-TDL, T1 ~ T4) for experiment demonstration. For QPSK/16QAM modulation, the 12.5-GBaud in-phase (I) and quadrature (Q) data signals are generated by a programmable digital-to-analog convertor (DAC), which is working at the 25-GHz clock. A frequency-doubler is used here after the 12.5-GHz source. The I/Q modulator is biased at the null point for signal modulation. The I/Q modulator is based on LiNbO3 waveguides with a modulation bandwidth of 27-GHz. One electronic phase shifter (PS) is used between the DAC clock signal and the RF source for comb generation, which synchronizes the modulation I/Q signals and the Nyquist pulses, keeping the peak of the Nyquist pulses within symbol durations.

To ensure the orthogonality as well as the de-correlation between tributaries, the delay line of each tributary is tunable and long enough. In our experiment, the tunable delay line consists of a fixed fiber (PM fiber jumpers, about 5-ns delay per meter) and a manually tunable time delay module (0 ~ 300 ps tunable delay). For the 3 channel Nyquist pulses multiplexing, we use a pair of 1:4 PM-OCs with two PM-TDLs. For the 5 channel Nyquist pulses time-division multiplexing, two 1:2 PM-OCs and two 1:4 PM-OCs are used with four PM-TDLs. The delayed time length is the integer multiples of the half zero-crossing duration (26.67 ps for the 37.5GBaud and 16 ps for 62.5GBaud Nyquist signal) to make sure the pulses are multiplexed at the zero-crossing time slots of adjacent pulses. There is about 125 symbols delay between each branch for decorrelation. After that, the PDM is realized by using a PM-OC to split the signals, a PM-TDL to provide over 100 symbols duration with pulse precisely aligned and a PBS for recombination. Then, the PDM all-optical Nyquist QPSK /16QAM signals with 37.5 and 62.5-GBaud are generated.

Figure 4 shows the eye-diagrams of the modulated Sinc-shaped Nyquist pulses and the time-division multiplexed all-optical Nyquist signals. The time scale of all these figures is 20 ps/div. Figure 4(a,c) are the *Sinc*-shaped Nyquist pulses of 3 × 12.5-GHz comb after 12.5-GBaud QPSK and 16QAM modulations, while 4(e) and 4(g) are the Nyquist pulses of 5 × 12.5-GHz comb after modulations. Clear eye-diagrams can be observed for the modulated pulses. Figure 4(b,d,f,g) shows the eye-diagrams of the TDM 37.5-GBaud QPSK, 37.5-GBaud 16QAM, 62.5-GBaud QPSK and 62.5-GBaud 16QAM. Since all the Nyquist pulses are multiplexed at the zero crossing points, orthogonal multiplexing in the time domain can be obtained without ISI. The Nyquist signals show characteristic amplitude over-shoots between the symbols (which are also the zero-crossing points of adjacent pulses) after the multiplexing.

The generated PDM all-optical signals are then launched into a re-circulating fiber loop, which consists of 5 spans of 80-km stand-single mode fiber (SSMF) with average loss of 18.5 dB and chromatic dispersion (CD) of 17 ps/km/nm, loop switches (SWs), optical coupler (OC), and Erbium-doped fiber amplifier (EDFA)-only amplification without optical dispersion compensation. One wavelength selective switch (WSS) is placed in the loop, which is programmed to work as an optical band-pass filter to suppress the ASE noise.

At the receiver side, a free-running ECL with linewidth less than 100 kHz is utilized as LO. A polarization-diversity 90° optical hybrid and four balanced photo-detectors (PDs) are used for coherent detection. The balanced PDs used in our experiment are from u2t with 50 GHz 3-dB bandwidth. At the receiver, the digital ADCs are working at 160 GSa/s with 65 GHz bandwidth as a real-time sampling oscilloscope (Lecroy, Labmaster 10-65Zi). After the ADC, the off-line digital signal processing is then applied for four channel sampled data sequence. The data is first resampled to 2 samples per symbol with time recovery, and then processed by the modified QPSK and 16QAM digital signal processing. For signals after fiber transmission, digital chromatic dispersion compensation^22^ is applied before clock recovery. Classical equalization such as constant modulus algorithm (CMA) and cascaded multi-modulus algorithm (CMMA) are used for QPSK and 16QAM, respectively. Since the phases between the symbols in each tributary are unknown, we need to do the time partitioning after the polarization demultiplexing but before carrier recovery. The frequency offset estimation (FOE) and phase recovery are applied for each TDM tributaries. It is different from the DSP in^12^ using continuous phase recovery, where an actively temperature controlled phase-stabilized (PS) multiplexer for OTDM is required. After equalization the bits of the individual tributaries are decoded and errors are counted together.

Figure 5 shows the measured back-to-back (BTB) bit-error-ratio (BER) of PDM all-optical Nyquist 37.5 GBaud QPSK/16QAM and 62.5 GBaud QPSK/16QAM signals versus the optical signal-to-noise ratio (OSNR) in 0.1-nm reference bandwidth. The OSNR is measured by using the optical spectrum analyzer (Yokogawa, AQ6370B) and the ASE noise is measured within 0.1 nm. In our experiment, the signals are sampled and stored by using a real-time sampling oscilloscope (Lecroy Labmaster 10–65Zi) with 160 GSa/s and the BER is measured by comparing with the original transmitted data pattern after the offline processing. The recording length is set longer enough for BER counting. The original transmitted data pattern is pseudo-random bit sequence with the length of 2^15^-1 for signal generation.The theory BER/OSNR curves of these signals are also plotted for reference.

There are less than 0.8-dB and 1.8-dB OSNR penalties relative to theoretical results for 37.5-GBaud PDM-QPSK and 16QAM signals at BER of 1 × 10^−3^. However, there are larger than 2.5-dB and 4-dB penalties relative to theoretical results for 62.5-GBaud PDM-QPSK and 16QAM signals at the same BER level. We believe the discrepancy between experimental and theoretical results is mainly due to the inaccurate time delays for TDM when using manually tunable delay lines. These time errors reduce the pulse orthogonality in time domain and cause ISI. Higher symbol rate and higher level modulation format signals are more sensitive. We believe better performance can be achieved by integrated signal generation and modulation setup. Inset (i) shows the spectrum of 37.5 and 62.5 GBaud PDM-16QAM (0.02-nm resolution). A better choice for signal generation is using high speed DACs, however, the bandwidth of commercial DACs is still limited. The proposed scheme in our paper provides on alternative method for high symbol rate Nyquist signal generation with good performances and high quality

We also measure the BER performance of the PDM all-optical Nyquist 37.5-GBaud 16QAM, 62.5-GBaud QPSK and 16QAM signals as a function of transmission distance as shown in Fig. 5. 4800-km transmission distance can be obtained for 62.5-GBaud PDM all-optical Nyquist QPSK signals below the BER of 2 × 10^−2^ (20% soft-decision FEC). 1200-km and 850-km can be achieved for the 37.5 and 62.5-GBaud 16QAM signals below the same BER level. Therefore, the generation, high level modulation formats, fiber-transmission and full-band coherent detection of PDM all-optical Nyquist signal is demonstrated by our experiment results.

### 25-GHz spacing combs and Nyquist pulses

The optical spectra of the generated 3 × 25-GHz and 5 × 25 GHz flat optical comb are shown in Fig. 6(a,b). The power difference of the generated 3-tone and 5-tone combs with 25 GHz carrier spacing after the filter is also less than 0.3 dB. The waveforms of the 3 and 5-tone combs are shown in Fig. 6(c,d) as Sinc-shaped pulses. We can see, for the 3 × 25-GHz comb, the Sinc-shaped Nyquist pulses have two zero-crossing points between each Nyquist pulse and the pulse repeating period is 40 ps. The repeating period is also 40 ps for the 5 × 25-GHz comb. However, the Nyquist pulses have four zero-crossing points between each pulse and the pulse durations between two zero-crossing point in (c) and (d) are 26.66 and 16 ps, respectively.

For Nyquist signal generation based on the combs with 25-GHz carrier spacing, we use the same scheme and experiment setup shown in Fig. 2 but with 25-GBaud signals generated by the DAC. The delay line of each tributary is adjusted with 125 symbols for the new Nyquist pulses. The PDM all-optical Nyquist QPSK signals with 75 and 125-GBaud are generated as shown in Fig. 7. Figure 7(a,b) shows the optical spectrum of 75 and 125-GBaud Nyquist QPSK signals, and the eye diagrams of 75 and 125-GBaud all optical Nyquist QPSK signals is shown in Fig. 7(c,d).

The BTB BER performance of the generated all-optical Nyquist 75 and 125-GBaud PDM-QPSK signals are measured versus the OSNR as shown in Fig. 8(a). Compared with theoretical BER curve, there is 2-dB OSNR penalty for Nyquist 75-GBaud PDM-QPSK signal, and about 4-dB penalty for the 125-GBaud PDM-QPSK signal. Both results in Figs 5 and 8 are better compared with^16^. Compared with the equipment used in^16^, in this paper we measure the time-domain waveforms by using the Agilent 86118A remote sampling module with 86107A precision timebase module. Jitter performance has been reduced by almost an order of magnitude to less than 200 fs RMS. Timebase resolution has also been improved from the 10 ps/division to 2 ps/division. Because the 86107A improves timebase jitter and linearity by more than a factor of five it provides extremely accurate views of high symbol rate signals. And we have confirmed that, this module can help us do more precise time-delay tuning. The amplitude measurement of the pulses is also more stable.

The transmission performance of generated 75 and 125-GBaud all optical Nyquist PDM-QPSK signals are shown in Fig. 8(b). Transmission distance of over 4000-km can be achieved for 75-GBaud all-optical Nyquist PDM-QPSK signal below the BER of 2 × 10^−2^ (20% soft-decision FEC). Over 2400-km can be achieved for the 125-GBaud QPSK signals below the same BER level. Therefore, the generation of high symbol rate signals with fiber-transmission and full-band coherent detection of PDM all-optical Nyquist signal is demonstrated by our experimental results.

### 1-Tb/s single-carrier PDM-16QAM all-optical Nyquist signal generation and detection

We have also experimentally demonstrated 1-Tb/s single-carrier all-optical Nyquist PDM-16QAM (125 GBaud) signal generation and full-band single-receiver coherent detection, which shows the advantage and feasibility of the single-carrier all-optical Nyquist signal. The transmitter setup of the 1-Tb/s all-optical Nyquist PDM-16QAM (125 GBaud) signal generation and full-band signal coherent detection is shown in Fig. 9(a), which is similar to that in Fig. 2. Inset (b) shows the 25-GHz carrier-spacing comb after the band-pass filter with peak power fluctuation less than 0.3 dB. After the PM-TOF, as shown in Fig. 9(c), the periodic sinc-shaped Nyquist pulses of 5 × 25-GHz combs are clearly obtained. The major difference is that the 25-GBaud 16QAM signals are generated by a commercial DAC at 64 GSa/s, the eye-diagram of the 4-level data signals is shown in (d). The clock of the DAC is synchronized with the RF driving signal for pulse generation. The periodic sinc-shaped pulse after the 25-GBaud 16QAM modulation is shown in Fig. 9(e). Finally, Fig. 9(f) shows the clear eye-diagram of the 125-GBaud orth-OTDM all-optical Nyquist 16QAM signal, and Fig. 9(g) shows the optical spectrum of 125-GBaud Nyquist16QAM signals.

The receiver setup is identical to that in Fig. 2. The back-to-back BER performance of the generated all-optical Nyquist 125-GBaud PDM-16QAM signal versus the OSNR is shown in Fig. 10(a). Compared with the theoretical BER curve, there is about 6.5-dB penalty for the 125-GBaud PDM-16QAM signal at the BER of 1 × 10^−2^ due to the inaccurate time delay and bandwidth limitation in the receiver. Figure 10(b) shows the BTB BER as a function of the symbol rate of all-optical Nyquist PDM-16QAM signals. Better performances can be achieved for lower-baud-rate signals. Therefore, a 1Tb/s single-carrier all-optical Nyquist PDM-16QAM (125 GBaud) signal is successfully generated and detected by one receiver. Assuming the use of 20% forward-error correction (FEC) at 2.0 × 10^−2^, the required OSNR is 29.5-dB and the demonstrated 125-GBaud all-optical Nyquist PDM-16QAM signal with 1-Tb/s line rate can provide a net information rate of 833.3 Gb/s.

## Discussion

Here in this paper, we extend our previous work and report the fully experimental demonstration of PDM all-optical Nyquist signal generation based on Sinc-shaped Nyquist pulse with advanced modulation formats, fiber-transmission and single-receiver full-band coherent detection. Different from the regular OTDM system with complicated demultiplexing setup, the polarization-multiplexed Nyquist signals are fully-band detected as one signal using a high speed digital coherent receiver with a free-running LO. Using this scheme, we have successfully demonstrated the generation, fiber transmission and single-receiver full-band coherent detection of all-optical Nyquist PDM-QPSK signals up to 125-GBaud, and all-optical Nyquist PDM-16QAM signals up to 62.5-GBaud. We also demonstrated the generation and coherent detection of all-optical Nyquist 125-GBaud PDM-16QAM signal at a line rate of 1 Tb/s based on periodic Sinc-shaped pulses and orth-OTDM. A net information rate of 833.3 Gb/s can be achieved assuming the use of 20% FEC.

Transmission on a single optical carrier using single transponder emerges as an attractive solution. Namely, minimizing the number of channel subcarriers reduces the number of deployed optical components, which typically determinate the transponder cost. There have been a lot of efforts made on this area these years, from device to system. However, the speed and bandwidth of DAC at the transmitter for signal generation is much less than the ADC at the receiver for signal detection these years. Lots of effort has been done in the transmitter side to increase the speed, such as ETDM^17^,^18^,^19^, OTDM^12^,^16^, super-channel or multi-carrier^7^ and multiple spectral slices synthesis multiplexing technology^13^,^14^, while keeping the single receiver with broadband components.

In our paper, the system is based on the all-optical Nyquist Sinc-shaped pulse generation and orthogonal time-division multiplexing, which makes it spectral-efficient as analyzed in^15^,^16^. In order to reduce the complexity in the receiver side, we treat the generated signal as one entire single-carrier signal and detect it with full-band information using a broadband receiver. The feasibility of high symbol-rate all-optical Nyquist signals generation, transmission and digital coherent detection is demonstrated.

## Methods

### Experimental measurement

The signals generation for 12.5-GBaud QPSK/16QAM and 25-GBaud QPSK is based on the DAC from MICRAM (VEGA DAC), working at 25-GSa/s using a 25-GHz external clock. The 25-GBaud 16QAM is generated by the Fujistu DAC, working at 64-GSa/s using an external 2-GHz clock. The I/Q modulator is with a modulation bandwidth of 27-GHz based on LiNbO3 waveguides. The optical spectra of the signals are all measured by a commercial optical spectrum analyzer (Yokogawa, AQ6370B). The time-domain waveforms of the Nyquist pulse and multiplexed signals are measured by an optical sampling oscilloscope using the Agilent 86118A remote sampling module with 86107A precision timebase module. At the receiver-side, the PDs are from u2t with 50 GHz 3-dB bandwidth, and the digital ADCs are working at 160GSa/s with 65GHz bandwidth as a real-time sampling oscilloscope (Lecroy, Labmaster 10-65Zi).

### Numerical Simulation and Analysis

**Figure 1:**
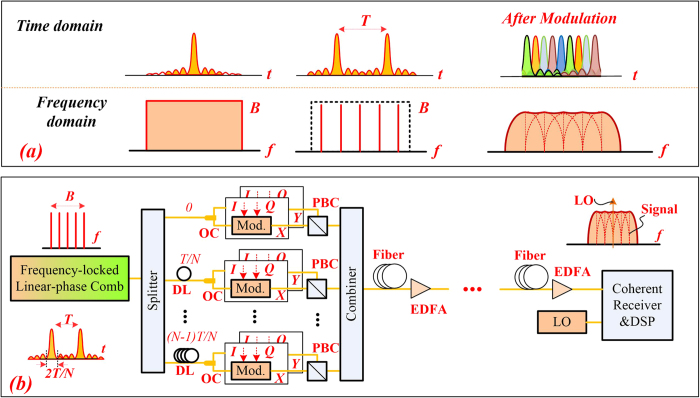
The basic concept and principle. (**a**) The time-frequency correspondence for sinc-shaped Nyquist pulses and the modulated Nyquist signals; (**b**) The principle of polarization-multiplexed all-optical Nyquist signal generation, modulation, multiplexing, fiber transmission and full-band coherent detection.

**Figure 2:**
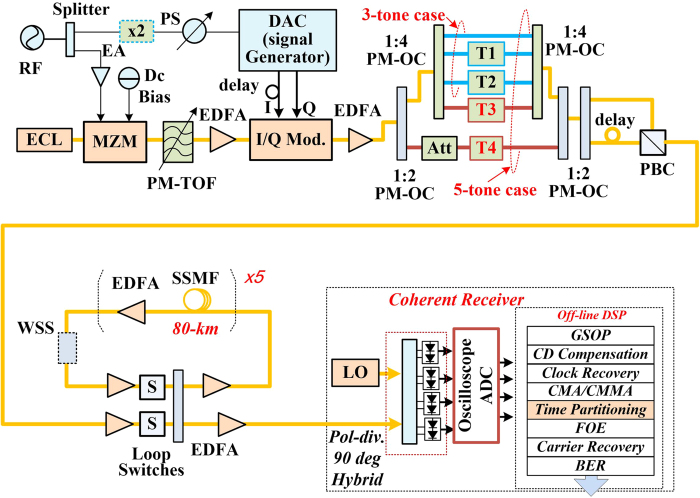
Experimental Setup of all-optical polarization multiplexed Nyquist signal generation, fiber transmission and full-band signal coherent detection. (RF: radio frequency; PS: phase shifter; DAC: digital-analog-convertor, working at 25-GHz clock; EA: electrical amplifier; ECL: external cavity laser; MZM: Mach–Zehnder modulator; I/Q Mod.: In-phase and quadrature modulator; PM-TOF: polarization-maintaining tunable optical filter; PM-OC: polarization-maintaining optical coupler; T1, T4: tunable optical delay-line consists of fixed fiber jumper and manually tunable optical time delay; ATT: tunable attenuator; PBC: polarization beam combiner; SSFM: stand single-mode fiber; S: switch; WSS: wavelength selective switch; EDFA: erbium doped fiber amplifier; LO: local oscillator).

**Figure 3:**
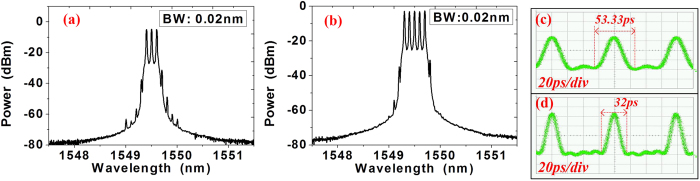
The spectrum and the pulse waveform. (**a**) is the optical spectra of the generated 3-tone comb with 12.5 GHz carrier spacing after the PM-TOF; (**c**) is the 5-tone comb with 12.5 GHz carrier spacing after PM-TOF. The resolution in (**a**) and (**b**) is 0.02-nm. (**c**) and (**d**) are the Sinc-shaped Nyquist pulses of the 3 × 12.5 and 5 × 12.5-GHz combs. The time scale of (**c**) and (**d**) is 20 ps/div.

**Figure 4:**
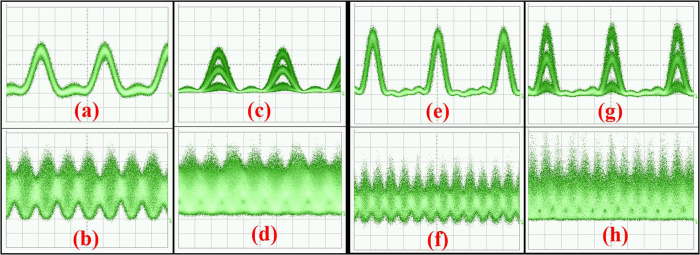
Eye diagrams. (**a,c**) are eye-diagrams of the Sinc-shaped Nyquist pulses of 3 × 12.5-GHz comb after 12.5-GBaud QPSK and 16QAM modulation; (**b,d**) are the eye-diagrams of the 37.5-GBaud all-optical Nyquist QPSK and 16QAM signals; (**e,g**) are the Sinc-shaped Nyquist pulses of the 5 × 12.5-GHz comb after modulation; (**f,h**) are the eye-diagrams of the 62.5-GBaud all-optical Nyquist QPSK and 16QAM signals. The time scale of all these figures is 20 ps/div.

To study the experimental implementation penalty caused by the inaccurate time-delays and power imbalance (power difference) of the tributaries for TDM when using manually tunable modules, we setup a numerical simulation model to test the impairments under different symbol rate and different level modulation formats. The numerical simulation system is also based on the Sinc-shaped Nyquist pulses generated by using the MZM driven by the RF as shown in Fig. 1.

We initially study the OSNR penalty caused by time offset due to the inaccurate time-delays. Since the tunable time-delay in our experiment is based on a manually tunable time delay module, incorrect time-delays can cause some time-offset to each pulse in the OTDM. Here, we considered three simple cases of time-offset as shown in Fig. 11. Fig. 11(a) is the case where only one pulse (the second pulse) placed is in incorrect time, with a time offset represented as delta_t1. The other two cases, shown in Fig. 11(b,c), are more complicated. The second and fourth pulse are assumed with same or opposite time offsets, represented as delta_t1 and delta_t2. In Fig. 11(b), delta_t1 = delta_t2 and in Fig. 11(c), delta_t1 = −delta_t2.

**Figure 5:**
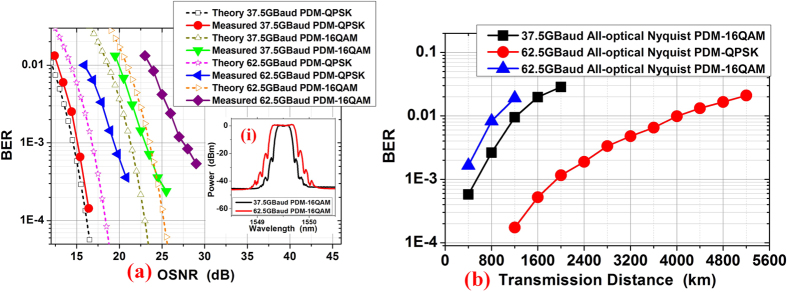
The BER results. (**a**) The back-to-back BER versus the OSNR for different signals comparing with theoretical curves. Inset (i) shows the spectrum of 37.5 and 62.5 GBaud PDM-16QAM (0.02-nm resolution). (**b**) BER performance versus transmission distance for the PDM all-optical Nyquist signals.

**Figure 6:**
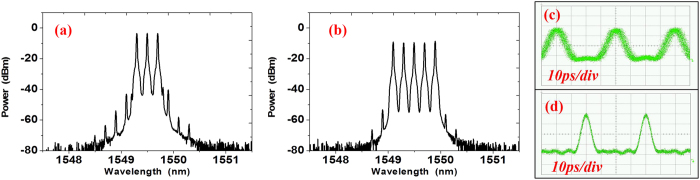
The spectrum and the pulse waveform. (**a**) the optical spectra of the generated 3-tone comb with 25-GHz carrier spacing after the PM-TOF; (**c**) the 5-tone comb with 25-GHz carrier spacing after PM-TOF. The resolution in (**a,b**) is 0.02-nm. (**c,d**) are the Sinc-shaped Nyquist pulses of the 3 × 25 and 5 × 25-GHz combs. The time scale of (**c,d**) is 10 ps/div.

**Figure 7:**
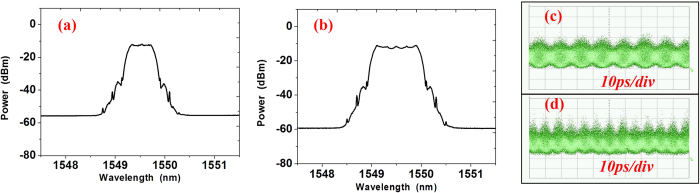
Optical spectra and eye-diagram of Nyquist signals. (**a,b**) are the optical spectrum of 75 and 125-GBaud all optical Nyquist signals; (**c,d**) are the eye-diagrams of 75 and 125-GBaud all optical Nyquist QPSK signals. The resolution in (**a,b**) is 0.02-nm.

**Figure 8:**
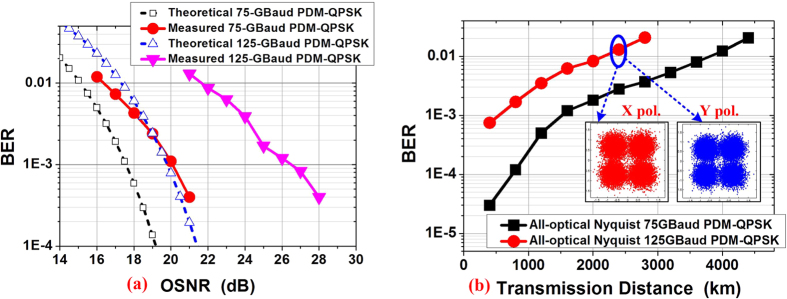
The BER results. (**a**) The BTB BER results versus the OSNR for 75 and 125-GBaud all-optical PDM-QPSK signals comparing with theoretical curves; (**b**) The transmission BER results of 75 and 125-GBaud all-optical PDM-QPSK signals versus the transmission distance.

**Figure 9:**
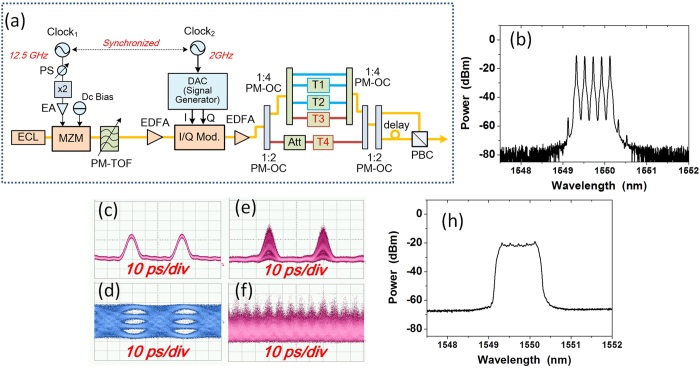
The experiment setup of 1-Tb/s all-optical Nyquist PDM-16QAM(125 GBaud) generation. Insets (**b**) are the 5 × 25-GHz comb, (**c**–**f**) are the eye-diagrams measured in 10 ps/div. (**c**) is the Sinc-shaped pulse before modulation, (**d**) is the four level signal generated by DAC, (**e**) is the Sinc-shaped pulse after modulation and (**f**) is the TDM signal of 125-GBaud 16QAM signals. (**h**) is the optical spectrum of signals after modulation. All the optical spectra are measured in 0.02 nm resolution.

**Figure 10:**
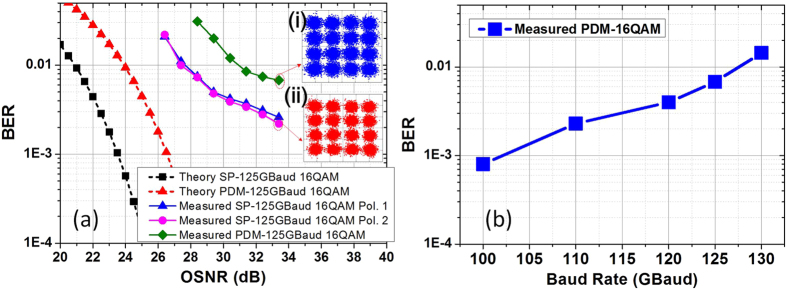
BER performances of the generated signals. (**a**) The back-to-back BER vs. OSNR for 125-GBaud all-optical Nyquist PDM-16QAM signal, constellations at (i) X and (ii) Y pol. In the PDM case at OSNR of 33.4 dB; (**b**) the BER vs. symbol rate for all-optical Nyquist PDM-16QAM signals.

**Figure 11:**
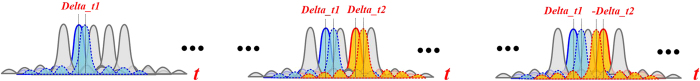
Time-delay offset in orthogonal time-division multiplexing (orth-OTDM). (**a**) Only one pulse with time-delay offset represented as delta_t1 (the second pulse); (**b**) two pulses with same position time-delay offset represented as delta_t1 and delta_t2, and delta_t1 = delta_t2; (**c**) two pulses with opposite position time-delay offset, represented as delta_t1 and –delta_2, and delta_t1 = −delta_t2.

**Figure 12:**
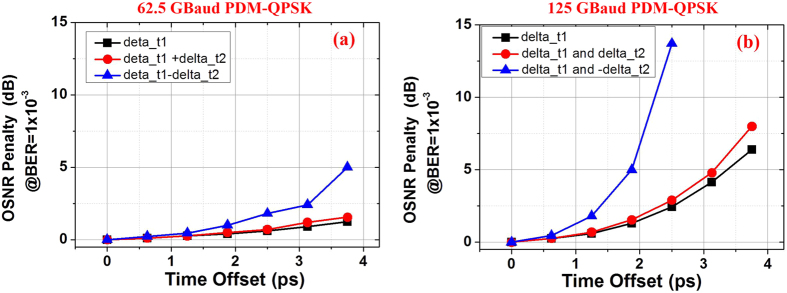
The OSNR penalty at BER of 1 × 10^−3^ as a function of time offset. (**a**) 62.5 and (**b**) 125 Gbaud PDM-QPSK signals are tested under the three different time offset cases in Fig. 9.

Two different symbol-rate signals are simulated and calculated here, including the 62.5 and 125-GBaud all-optical Nyquist PDM-QPSK signals. Both signals are generated based on the proposed schemes, using 5 × 12.5 and 5 × 25-GHz combs. The OSNR penalty at BER of 1 × 10^−3^ as a function of time-offset for 62.5 and 125-GBaud signals under the three offset cases in Fig. 11 are shown in Fig. 12(a,b), respectively. Several conclusions can be made by these results in Fig. 12. First, both Fig. 12(a,b) show that the OSNR penalty increases with the addition of time-offset, which is due to the increased inter-symbol-interference (ISI) of adjacent symbols. Second, the opposite time-offset of the different pulses shows larger OSNR penalty compared with the other two cases, because the opposite time-offsets cause more severe ISI to particular symbols (such as the symbols of the third pulse). Finally, as proved by the experimental results above, higher symbol-rate signal is more sensitive to the time-offset compared with lower symbol-rate signal. Since lower symbol-rate signals have longer symbol duration, they are less sensitive to the time-offset. They are with good agreement with the results in Figs 5 and 8.

**Figure 13:**
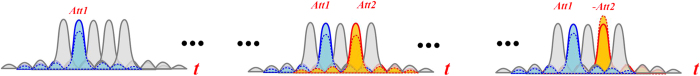
Pulse power differences in orthogonal time-division multiplexing (orth-OTDM). (**a**) Only one pulse with power difference represented as Att1 (the second pulse); (**b**) two pulses with same power differences represented as Att1 and Att2, and Att1 = Att2; (**c**) two pulses with opposite power differences, represented as Att1 and −Att2, and Att1 = −Att2.

We also study the impact of power differences of the pulses in the different tributaries for TDM. Since the receiver-side adaptive equalization is based on the identical average-power of each pulse, the power imbalance can cause large penalty due to the error fluctuations in the convergence process of the taps of adaptive equalizers. Similar to the cases in Fig. 11, we also test three different cases as shown in Fig. 13. Figure 13(a) is the case where only one pulse (the second pulse) has smaller power difference, represented as Att1. The other two cases, shown in Fig. 11(b,c), are more complicated. The second and fourth pulse are assumed with same or opposite power differences, represented as Att1 and Att2. In Fig. 13 (b), Att1 = Att2 and in Fig. 13(c), Att1 = −Att2. When the attenuation value is negative, it means the pulse has larger average power.

**Figure 14:**
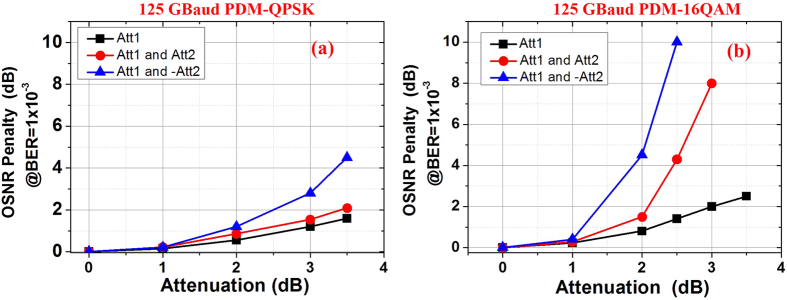
The OSNR penalty at BER of 1 × 10^−3^ as a function of pulse power difference. (**a**) 125-GBaud PDM-QPSK and (**b**) 125-Gbaud PDM-16QAM signals are tested under the three different power difference cases in Fig. 11.

Under these three cases, two different modulation formats are simulated and tested here, including the 125-GBaud all-optical Nyquist PDM-QPSK and PDM-16QAM signals. Again, both simulated signals are generated based on the proposed schemes, using the 5 × 25-GHz combs. The OSNR penalty at BER of 1 × 10^−3^ as a function of power differences for 125-GBaud PDM-QPSK and PDM-16QAM signals under the three power-difference cases in Fig. 13 are shown in Fig. 14(a,b), respectively. We can observe that the OSNR penalty increases with the addition of power difference, as shown in both Fig. 14(a,b). We believe that the penalty is due to the error fluctuations in the convergence process of the taps of adaptive equalizers under power imbalance cases. On the other hand, the opposite power difference case shows larger OSNR penalty compared with the other two cases. This is because the opposite power differences produce doubled power imbalance overall. Finally, we can see that higher level symbol-format (PDM-16QAM) is much more sensitive to the power difference compared with lower symbol-rate signal. It is because that multi-level or multi-modulus equalization is used for higher level modulation formats, the average power differences between each symbol causes larger decision error, which have much larger impact on the taps convergence. Experiment results in Figs 8 and 5 also confirm this conclusion.

### Transmission Link Analysis

The re-circulating fiber loop, consists of 5 spans of 80-km stand-single mode fiber (SSMF) with average loss of 18.5 dB per span and chromatic dispersion (CD) of 17 ps/km/nm, loop switches (SWs), optical coupler (OC), and Erbium-doped fiber amplifier (EDFA)-only amplification without optical dispersion compensation. One wavelength selective switch (WSS) is placed in the loop, which is programmed to work as an optical band-pass filter to suppress the ASE noise. Since coherent detection is used here, the fiber dispersion is compensated at the receiver by using digital frequency-domain CD compensation algorithm. Therefore, the deterioration of the transmission link is mainly caused by two factors: the first is the increasing of noise or the decreasing of the OSNR caused by the loss of fiber and EDFA noise figure; the second, which is more important for long haul transmission, is the fiber Kerr nonlinearity impairment.

As analyzed in^24^, the OSNR along with the fiber transmission link based on distributed amplification (such as EDFA) can be predicated by the following equations:
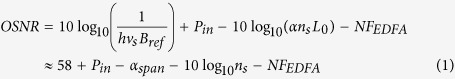
here *hv*_*s*_ is the photon energy at signal wavelength, *B*_*ref*_ is the reference bandwidth of OSNR, which is ~12.5-Ghz, *P*_*in*_ is the fiber launch power (in dBm), the *α*_*span*_ is the fiber loss per span, and *n*_*s*_ is the fiber span number, and *NF*_*EDFA*_ is the noise figure of the EDFA. Therefore, increasing the fiber length or fiber span number, the OSNR will decrease and noise is increased. Although by increasing the launch power or input power at the transmitter can increase the OSNR after transmission, however, the other key issue, Kerr fiber nonlinearity, arises.

Generally, the nonlinear phase distortion of fiber can be simply expressed as
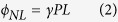
here *γ* is the nonlinear parameter, *P* is the signal power and *L* is the fiber length. Therefore, for long-haul transmission, especially for the distributed amplification system, the fiber nonlinearity is a key issue. In this way, the transmission results departs from the Shannon limit due to the Kerr fiber nonlinearity impairment in long haul transmission^24^,^25^.

## Additional Information

**How to cite this article**: Zhang, J. *et al.* Transmission and full-band coherent detection of polarization-multiplexed all-optical Nyquist signals generated by *Sinc*-shaped Nyquist pulses. *Sci. Rep.* **5**, 13649; doi: 10.1038/srep13649 (2015).
